# The Role of Gut Microbiota, Nutrition, and Physical Activity in Depression and Obesity—Interdependent Mechanisms/Co-Occurrence

**DOI:** 10.3390/nu16071039

**Published:** 2024-04-02

**Authors:** Klaudia Sochacka, Agata Kotowska, Sabina Lachowicz-Wiśniewska

**Affiliations:** 1 Faculty of Medicine and Health Sciences, Calisia University, 62-800 Kalisz, Poland; k.sochacka@uniwersytetkaliski.edu.pl; 2 Department of Social Policy, Institute of Sociological Sciences, College of Social Sciences, University of Rzeszow, 35-310 Rzeszow, Poland; akotowska@ur.edu.pl

**Keywords:** probiotic microorganisms, nutrients, depression, obesity

## Abstract

Obesity and depression are interdependent pathological disorders with strong inflammatory effects commonly found worldwide. They determine the health status of the population and cause key problems in terms of morbidity and mortality. The role of gut microbiota and its composition in the treatment of obesity and psychological factors is increasingly emphasized. Published research suggests that prebiotic, probiotic, or symbiotic preparations can effectively intervene in obesity treatment and mood-dysregulation alleviation. Thus, this literature review aims to highlight the role of intestinal microbiota in treating depression and obesity. An additional purpose is to indicate probiotics, including psychobiotics and prebiotics, potentially beneficial in supporting the treatment of these two diseases.

## 1. Introduction

According to the latest data from the World Health Organization (WHO), as many as 300 million people are affected by mental disorders [1]. Approximately 800,000 suicides per year are attributed to depression, making it one of the most prevalent mental illnesses. [1]. It is characterized by persistent feelings of sadness; lowered self-esteem; feelings of uselessness; excessive and unwarranted feelings of guilt; a decreased ability to concentrate, pay attention, and remember; and a significant loss of previous interests. The American Psychiatric Association includes depressive disorders in the Diagnostic and Statistical Manual of Mental Disorders 5 (DSM-5), a classification used to diagnose or rule out psychiatric disorders [2]. According to the DSM-5, depressive disorders encompass various classifications, including major depressive disorder, persistent depressive disorder (dysthymia), premenstrual dysphoric disorder, substance- or drug-induced depressive disorder, and depressive disorder due to a general medical condition, among others [3]. A common feature of all depressive disorders is a chronic depressed mood and irritability, accompanied by co-occurring somatic and cognitive changes. Consequently, they significantly reduce a person’s quality of life and daily functioning [2]. Depression is a significant risk factor for numerous metabolic diseases, including diabetes, obesity, and chronic heart disease. It is a fact that eating habits have a significant impact on mental health. Even single contingencies in a people’s life can influence their dietary preferences and choices, thereby correlating with their overall nutritional status, which will consequently affect their overall mental state [1]. Consuming a high-fat diet has been demonstrated to cause inflammation and obesity throughout the body [4].

On the other hand, obesity, defined by an excessive accumulation of body fat, has long been classified as a disease by the WHO and is listed in the International Classification of Diseases (ICD-10) as E66. It is determined by body mass index (BMI) and percentage of body fat. Furthermore, obesity poses a significant risk for several other diseases, including, but not limited to, cardiovascular disease, insulin resistance, neuropathy, and cancer. Age, gender, smoking, and chronic consumption of high-fat and highly processed diets, i.e., Western diets (WD), are the main predisposing factors for obesity. As an aside, it is worth noting that, despite the above classification, in the public perception, obesity is often treated solely as a consequence of an irresponsible lifestyle, and its control is not associated with the need for treatment, but only the demonstration of self-discipline by those affected. A stigmatizing and dismissive approach to obesity as an ‘aesthetic affliction’ can lead to the exacerbation of the pathological psychological conditions that accompany it in sufferers. It should be stressed that depression, anxiety disorders, and obesity very often co-exist and are interdependent. This is likely because these diseases share a common biological foundation. Depressive states often lead to heightened appetite and emotional regulation through food. When coupled with physical inactivity, this can result in a substantial weight gain. Moreover, due to the lack of sufficient public awareness of obesity, psychological problems often arise in people struggling with this condition, often leading to the emergence of depressive anxiety disorders. Numerous studies affirm a robust correlation between the simultaneous presence of obesity and depression [5]. Depression and obesity are considered to be among the biggest health problems in the world, both because of their prevalence and the high mortality rate from these diseases. The potential association between depression and obesity has been extensively studied and reported in the literature, although many relationships remain unclear. Both conditions pose an elevated risk of cardiovascular disease [6].

Recently, there has been a growing body of research aimed at comprehending the influence of the gut microbiota on energy storage and metabolism in the body. The microbiota exhibits significant differences between lean and obese individuals. In overweight individuals, it is characterized by a decrease in diversity and a significant reduction in commensal bacteria characterized by anti-inflammatory effects, while pathogenic microorganisms increase.

This leads to the manifestation of various metabolic pathways, which may be one of the etiological factors of obesity. Probiotic and prebiotic treatments are currently being used to restore the desired composition of the intestinal ecosystem [7].

Emotional stress is frequently experienced by obese patients, potentially resulting in mood disorders (MDs) such as depression and anxiety [8]. The risk of obesity was found to be 37% higher in patients with MD, while the risk of depression was 18% higher in obese patients, indicating that there may be a bidirectional relationship between obesity and MD [9]. A current review of *in vivo* studies on an animal model (mice) shows that obesity induced by a high-fat diet is responsible for the emergence of psychiatric conditions (anxiety and depression) [10]. Researchers are fascinated by the gut–brain axis because it plays a pivotal role in maintaining both physical and mental well-being. Disturbances in the gut microbiota’s composition may affect the gut–brain axis, leading to alterations in the individual’s mental and physical health [11].

Any abnormality in the composition of the gut microbiota, known as gut dysbiosis, can induce impaired energy utilization, leading to obesity, as the gut microbiota plays a crucial role in disposition and energy expenditure [12]. As mentioned earlier, the gut microbiota of lean and obese individuals differs significantly. Changes in the abundance of Bacteroidetes and Firmicutes suggest a significant role in mental health regulation [13]. Dysbiosis and gut inflammation have been associated with various mental illnesses, such as anxiety and depressive behavior, which are becoming increasingly prevalent in our society. Due to their ability to restore the proper balance of the gut microbiota, probiotics and psychobiotics might have a role in the treatment and prevention of anxiety and depression [14].

For moderate-to-severe depression, psychotherapy, including cognitive–behavioral therapy, meditation, and interpersonal psychotherapy, as well as pharmacological treatment, such as serotonin–norepinephrine reuptake inhibitors and selective serotonin reuptake inhibitors, are employed. There are several approved treatments, including antidepressants, atypical antidepressants, and tricyclic antidepressants [15]. Antidepressants, despite their proven efficacy, can cause problems due to significant weight increase. A cohort study with 294,719 participants indicated that antidepressants might contribute to a population-level weight gain of over 5% in the long term [16]. Alongside the therapies mentioned earlier, natural and alternative approaches like herbal medicine, physical activity, or meditation are also utilized [17]. A balanced diet following a rational eating pattern, stress reduction, and adequate sleep are also recommended [18]. Individuals grappling with depression exhibit decreased levels of vitamin B12 and D3 in contrast to those who are healthy [19]. Therefore, additional dietary supplementation is entirely warranted and may prove helpful in alleviating the symptoms of depression [20]. Innovative approaches to the treatment of depression, especially metabolic depression, suggest the administration of probiotics, including psychobiotics [15,21]. The gut microbiota may impact behavioral, physiological, and cognitive brain functions [22], suggesting that probiotics, including psychobiotics and their metabolites, could influence depression treatment [15]. Therefore, this review aims to underscore the role of intestinal microbiota in addressing depression and obesity. An additional purpose is to indicate that probiotics, including psychobiotics and prebiotics, are potentially beneficial in supporting the treatment of these two diseases.

## 2. The Role of Intestinal Microbiota

The human gastrointestinal tract hosts a complex ecosystem of microorganisms that profoundly affect the host and maintain overall organismal homeostasis. Several factors influence the development of human gut microbiota during infancy, with diet being the most significant, shaping its composition throughout life. Gut bacteria play crucial roles in maintaining immune and metabolic balance and defending against pathogens. Changes in gut bacteria composition (dysbiosis) are linked to various inflammatory diseases and infections [23]. A healthy human gut microbiota comprises a diverse microbial community of around 5000 species and 7000 strains, predominantly consisting of the bacterial clusters *Firmicutes*, *Bacteroidetes*, *Actinobacteria*, and *Proteobacteria* [24]. Recent studies highlight a close association between gut microbiota and the brain [25]. Probiotics, including psychobiotics, have emerged as potential treatments for psychiatric disorders and metabolic issues [26], suggesting their importance in mental disorder management [27,28]. Probiotics, consisting of live microorganisms offering health benefits to the host when consumed in adequate amounts, mainly belong to genera like *Lactobacillus*, *Bifidobacterium*, and *Saccharomyces* [29]. Psychobiotics, targeting mental health, are a subset of probiotics [30]. Probiotic microorganisms regulate the immune system, produce short-chain fatty acids (SCFAs), and bolster intestinal barrier integrity [27]. Various studies highlight the diversity and specificity of probiotic strains in influencing brain function. A meta-analysis by Miryam de Souza et al. [31] revealed that probiotics significantly alleviate depressive symptoms among individuals with depression [31]. Another study by Messaoudi et al. [32] found that consuming psychobiotics containing *Lactobacillus helveticus* R0052 and *Bifidobacterium longum* R0175 eased depression and anxiety symptoms compared to a placebo group [32]. Probiotics, including psychobiotics, can produce essential vitamins like B vitamins, crucial in depression etiology and treatment [28,29]. Moreover, probiotics serve as SCFA sources, which play vital roles in anti-inflammatory processes, insulin sensitivity enhancement, and fat accumulation reduction [33,34]. Probiotics, especially psychobiotics, are deemed significant adjunctive therapies for depression [26]. Skonieczna-Żydecka et al. [35] demonstrated that select probiotic strains directly activate neural pathways, regulate neurotrophic factors, and mitigate stress, including *Lactobacillus fermentum* NS8 and NS9, *Lactobacillus gasseri* OLL2809, and *Lactobacillus casei Shirota,* among others [35]. These strains significantly reduced depressive symptoms after four weeks of probiotic therapy [35]. In another study, patients consuming probiotics for eight weeks showed a greater improvement in well-being, as measured by a decrease in the Beck Depression Inventory scale [36]. Approximately 57% of gut microbiota composition is influenced by diet, while only 12% is due to genetic variation [37]. Probiotic interventions prevent and alleviate dysbiosis by enhancing the cytoskeletal structure, mucin secretion, and tight junction protein phosphorylation in the intestinal barrier, fostering improved gut microbiota variability and bacteriocin and organic acid production [38,39]. Probiotic and prebiotic preparations offer effective means to influence gut microbiota composition positively. Numerous types of bacteria, particularly psychobiotics, have been identified for their beneficial effects on mental health.

In a separate study, 40 patients received either a placebo or probiotics for 8 weeks, consisting of *Lactobacillus acidophilus* 2 × 10^9^ CFU (colony-forming unit), *Bifidobacteria bifidum*, and *Lactobacillus casei* 2 × 10^9^ CFU. The probiotic group exhibited a 5.7-point decrease on the Beck Depression Inventory scale (BDI scale), compared to a 1.5-point decrease in the placebo group, indicating a more significant improvement in well-being [36].

Approximately 57% of gut microbiota composition is influenced by diet, while only 12% is attributed to genetic variation [37]. Probiotic intervention has been shown in various studies to prevent and alleviate dysbiosis by enhancing the cytoskeletal structure, increasing mucin secretion through the activation of secretory proteins Mucin 2 and Mucin 3, and phosphorylating tight junction proteins in the intestinal barrier [38]. Improved dysbiosis is achieved through increased intestinal microbiota variability, and bacteriocin and organic acid production, creating an unfavorable environment for the colonization of pathogenic bacteria and their metabolites [39]. It is not only the composition of the intestinal microbiota that is essential but also the ability to alter it. This can be achieved with probiotic and prebiotic preparations. Research indicates that many types of bacteria, particularly psychobiotics, have a positive effect on mental health [40]. Their action influences the secretion of neuroactive substances such as GABA, also known as gamma-aminobutyric acid, a neurotransmitter with an inhibitory effect that moderates the stress response, and serotonin. Additionally, microbes have been shown to affect the HPA axis (hypothalamic–pituitary–adrenal axis), which exacerbates depressive symptoms and anxiety. Moreover, psychobiotics increase the concentration of oxytocin in the body, reducing the activity of the HPA axis and contributing to the regulation of the stress response and reduction of anxiety [41]. These include *Lactobacillus plantarum* strains 299V and PS128 (its long-term use has a beneficial effect on mood in depression (demonstrated by the Beck Depression Scale—BDI). On the other hand, *Bifidobacterium infantis* has a beneficial effect on regulating the HPA axis [42]. Gut microbes can produce a variety of neurotransmitters, acting both locally (in the gut) and affecting brain activity. *Bifidobacterium infantis* bacteria can increase plasma concentrations of tryptophan, which is a precursor of serotonin—a key neurotransmitter in regulating mood, appetite, and gastrointestinal function [39]. Strains such as *Lactobacillus brevis*, *Bifidobacterium dentium*, *Pseudomonas*, and *Escherichia coli* are capable of producing GABA; *Escheridia*, *Saccharomyces*, and *Bacillus* can manufacture norepinephrine; *Lactobacillus*—acetylcholine; *Enterococcus*, *Candida*, *Escheridia,* and *Streptococcus*—serotonin; and *Bacillus*—dopamine. The hypothesis that neurotransmitter deficiencies cause mood disorders, based on new data, may be linked to changes in microbial populations during intestinal dysbiosis [43,44].

The assessment of efficacy and the introduction of a new method to assist in the treatment of depression through probiotic and prebiotic therapy are promising, despite requiring numerous studies and meta-analyses for validation.

Among the most commonly studied strains are bacteria of the genus *Lactobacillus*. *In vivo* studies allow us to conclude that the application to rats subjected to the stress *L. helveticus* NS8 in an immobilization test resulted in a reduction in aggressive and depressive behavior. Blood corticosterone levels decreased, while serotonin and norepinephrine levels in the hippocampus increased [45]. A comparable research study was conducted by other authors [46], this time focusing on the effect of *L. reuteri* on mice exposed to chronic stress resulting from social failure. Improvements in depressive behavior were observed, potentially linked to an increase in 5-hydroxytryptamine (5-HT, or serotonin) [46]. Potential applications in the treatment of depression also include strains from the genus *Bifidobacteria*. One of the first studies was to test the effect of strain *B. infantis* 35,624 on depressive behavior induced by the swimming test in mice. The results showed that probiotic administration increased brainstem norepinephrine extent and decreased the manufacture of inflammatory cytokines—mainly IL-6 [47]. In another study, the administration of *B. infantis* led to an increased expression of tryptophan hydroxylase 1 (TPH1), resulting in the enhanced production of tryptophan, which serves as a precursor to serotonin. Others authors [48] developed a study demonstrating the synergistic antidepressant effects of bacteria from the above two genera. They investigated the effects of *L. mucosae* and *B. longum* on rats with stress-induced depression, and the findings indicated that the joint application of both strains led to a notable decrease in depressive symptoms, as evidenced by reductions in stress hormone levels, lipopolysaccharides, IL-6, and TNF-α [48]. A relatively new finding is the antidepressant and anti-anxiety effect of *Faecalibacterium prausnitzii* on chronically stressed mice. The research indicated that reduced levels of these bacteria in the gut microbiome correlated with increased depression severity and diminished mood. The positive impacts of this particular strain are facilitated by suppressing the nuclear factor NF-κB inflammatory pathway (a key factor in immune and inflammatory responses) through the release of anti-inflammatory microvesicles. Moreover, a decrease in inflammatory cytokine concentrations and fortification of the intestinal barrier (facilitated by butyrate, generated by the evaluated strain) were noted [49]. An antidepressant effect was also observed when treating *Clostridium butyricum* chronically stressed mice. The combination of this strain with antidepressants alleviates the symptoms of the disease in about 70% of cases [50].

Satisfactory results in animal models made it possible to conduct clinical trials in humans. In a psychiatric hospital in Switzerland, individuals diagnosed with depression were split into two cohorts. One group was administered a daily probiotic supplement comprising eight diverse bacterial strains, predominantly from the *Bifidobacteria* and *Lactobacillus* genera, while the other group received a placebo. Following a four-week period, the outcomes were assessed, revealing a notable enhancement in memory among patients consuming the probiotics. The deactivation of the hippocampus (the part of the brain that is primarily responsible for long-term and spatial memory), which is usually hyperactive in depressed patients, also had a beneficial effect on improving cognitive function. However, it should be mentioned that the study described here involved a small number of patients, due to the exclusion of cases with low compliance, thus requiring verification of the results in subsequent studies [51]. The effect of probiotics on depressive behavior proved to be of great interest, so a number of studies were conducted on different groups of people. The study group consisted of healthy patients and people with depressive symptoms, including elderly people and students, as well as patients with and without comorbidities. They received probiotic supplements with a variety of ingredients, but mainly from the genera *Bifidobacteria* and *Lactobacillus*. The efficacy of probiotics in mitigating the risk of severe depression has been broadly demonstrated, yet the findings remain inconclusive. In healthy individuals, no significant effect on emotion regulation was observed, making it difficult to confirm a beneficial effect of probiotics on mood disorders. Patient age also had a significant effect on the test results. A significant antidepressant effect was only observed in participants under 60 years of age. This shows that the composition of the probiotic used should be age appropriate. Importantly, studies presented that the use of a significant quantity of different strains is more effective than the use of a single strain [52]. Modulating the composition of the human microbiome cannot be achieved solely through probiotic supplementation. An approach involves fecal microbiota transplantation (FMT), where stool from a healthy donor is transferred into the gastrointestinal tract of an ailing recipient.

This method is often used for recurrent antibiotic-resistant *Clostridium difficile* infections. Research is currently being conducted into the use of fecal transplants to treat neurological conditions. In 2012, improvement after FMT was observed in 70% of patients with chronic fatigue syndrome, i.e., individuals diagnosed with irritable bowel syndrome (IBS). However, the number of ongoing studies is too small to conclusively confirm the beneficial effects of FMT on mood disorders [53].

Diseases of civilization, which include obesity, are currently a problem that we cannot cope with. Studies have highlighted the significance of probiotics and prebiotics in managing conditions like obesity. Research suggests that probiotics and synbiotics can have positive impacts on body mass index and fat mass in obese individuals, as well as for conditions such as insulin resistance, type 2 diabetes, and nonalcoholic fatty liver disease (NAFLD) [54].

Over the past decade, there has been a notable rise in the utilization of probiotics and synbiotics for both preventive and therapeutic purposes. These ingredients, commonly found in functional foods and nutraceuticals, offer health advantages by modulating the microbial ecosystem and enhancing gut barrier integrity. Obesity stands as a significant global health concern, impacting nations across the spectrum, from developed to emerging economies. Defined by an abnormal accumulation of white adipose tissue, it serves as a primary contributor to the onset of conditions such as diabetes, cardiovascular ailments, and cancer. In a study involving obese adolescents, the impact of *Lactobacillus salivarius* Ls-33 on gut microbiota, anthropometric parameters, inflammatory markers, and lipid and carbohydrate metabolism was examined. Following the administration of *L. salivarius* Ls-33, there was a notable increase in the ratio of bacteria from the *Bacteroides* and *Porphyromonas* groups compared to bacteria from the *Firmicutes* group, encompassing *Blautia coccoides*, *Clostridium* XIV, *Roseburia*, and the Eubacterium rectale group. However, no significant changes in body measurements or reduction in inflammation were observed [55,56]. In 2010, findings from a randomized clinical trial assessing the effectiveness of probiotics as a supplementary therapy for overweight and obesity were released. The impact of *L. gasseri* SBT2055 on alterations in anthropometric measurements was investigated in 43 individuals with a BMI ranging from 24.2 to 30.7 kg/m^2^ and visceral obesity. Patients in the study group consumed kefir enriched with the bacterial strain described above for 12 weeks, while the control group consisted of 44 obese patients who did not consume probiotics in their diet. Observational findings suggest that probiotic supplementation leads to reductions in body weight, BMI, waist, and hip circumference, as well as visceral fat [57]. The effects of administering *L. acidophilus* La5, *L. casei* DN001, and *B. lactis* Bb12 were assessed in high-BMI individuals, who were randomly divided into three groups based on the respective intervention diets: one group consumed plain yoghurt with a low-calorie diet (RLCD), the second group consumed probiotic yoghurt with a low-calorie diet (PLCD), and the third group consumed probiotic yoghurt without a low-calorie diet (PWLCD) for approximately two months. A reduction in BMI, percentage body fat, and leptin levels was observed, with more pronounced effects in the groups following a weight-loss diet containing probiotic yoghurt. Additionally, after the 8-week intervention, a more significant reduction in serum CRP levels was observed in the PWLCD group compared to the PLCD and RLCD groups [58]. The impact of *L. rhamnosus* CGMCC1.3724 combined with oligo-fructose and inulin supplementation on weight loss and maintenance was investigated in obese men and women over 24 weeks. Women in the *L. rhamnosus* group experienced significantly greater weight loss after the first 12 weeks compared to those in the placebo group, while there was no significant difference in men between the two groups. Weight loss induced by *L. rhamnosus* in women was associated not only with a significant decrease in fat mass and circulating leptin levels (a hormone involved in regulating hunger and satiety) but also with changes in the relative abundance of bacteria from the *Lachnospiraceae* family in their stools—this family belongs to the *Firmicutes* phylum, which has previously been associated with obesity [59,60]. In recent years, there has been a growing focus on incorporating probiotics as a supplementary therapy for managing conditions such as obesity or type 2 diabetes. An interesting perspective is the so-called new-generation probiotics, i.e., probiotic preparations based on unique strains isolated from the human intestine, with high therapeutic potential. Their representative is the commensal bacterium *Akkermansia muciniphila*, whose numbers in the intestines of healthy people are significantly higher than those of patients with metabolic disorders. Scientific studies have shown that this bacterium has positive effects, including the ability to increase the intestinal barrier, stimulate the host immune system, suppress inflammation, or modify the metabolic response [61]. Obese mice and those on a high-fat diet (HFD) exhibited a notable decrease in the overall abundance of *A. muciniphila* compared to lean counterparts, with a negative correlation observed with fat mass [62]. Initial *in vivo* investigations in rodents provided evidence of the metabolic impacts of *A. muciniphila*. These findings highlighted a link between the microbial composition and the onset of obesity and type 2 diabetes, particularly noting a progressive decline in *A. muciniphila* levels in mice fed a high-fat diet [63]. Supplementation with *A. muciniphila* has been shown to reverse metabolic abnormalities caused by this diet, including fat gain, endotoxemia, and insulin resistance. Although *A. muciniphila* colonization did not alter serum cholesterol and triglyceride levels, it improved atherosclerosis [64,65].

Furthermore, *in vitro*, as well as *in vivo*, studies, while valuable for initial investigations, have limitations when extrapolating findings to human populations due to differences in physiology, metabolism, and genetic factors. Therefore, results observed in animal models may not always directly translate to humans. Furthermore, individual responses to probiotics can vary significantly due to factors such as age, gender, genetics, diet, gut microbiota composition, and overall health status. What works for one person may not necessarily work for another, making it challenging to predict the effectiveness of probiotics universally [66]. As a result, there is a critical need for further research to establish the efficacy of probiotics in humans through well-designed clinical trials. These trials should consider various factors, such as probiotic strain specificity, dosage, duration of treatment, and the target population’s characteristics. Moreover, it is essential to recognize that probiotic interventions are not standalone solutions but should be considered within the context of overall dietary and lifestyle factors. Factors such as diet quality, physical activity, stress levels, and sleep patterns can influence gut health and microbial balance, affecting the effectiveness of probiotics [67]. Therefore, a holistic approach that integrates probiotic interventions with dietary modifications and lifestyle changes may yield more significant benefits for improving gut health and overall well-being. This underscores the importance of personalized and multifaceted approaches in leveraging the potential of probiotics for health promotion and disease prevention.

## 3. The Influence of Dietary Patterns on Mental Well-Being, Particularly in Alleviating Depressive Symptoms

The brain uses a significant proportion of the energy and nutrients it obtains. Its good nutrition requires an adequate supply of amino acids, fats, vitamins, minerals, and trace elements. The relationship between depression and dietary habits has been well documented [68]. An integral component among these is the antioxidant mechanism, which is crucial in the onset of mental disorders and reliant on the presence of nutrients inherent in our diet [69]. Likewise, the level of brain-derived neurotrophic factor (BDNF), vital for neuroplasticity and regeneration, hinges on nutrient intake [70]. Studies have revealed that adopting healthy dietary patterns correlates with reduced instances of depression and suicide. Clinical trials have explored the efficacy of dietary modifications as a therapeutic approach for depression [71]. Certain nutrient supplements may offer benefits in managing psychiatric conditions. These include compounds like S-adenosylmethionine; *N*-acetylcysteine; zinc; and B vitamins, including folic acid, as well as vitamin D. Omega-3 polyunsaturated fatty acids, in particular, have diverse effects, participating in synaptogenesis, modulating receptor activity, exerting anti-inflammatory properties, and impacting neurotransmitter regulation [72]. Zinc deficiency has been linked to heightened depressive symptoms, and zinc supplementation alongside antidepressant therapy aids in mood stabilization. Zinc also regulates cytokine function and influences neurogenesis by modulating BDNF levels. Vitamin B is essential for neural tissue function. Deficiency in folic acid (vitamin B9) has been associated with depressive symptoms and observed in individuals with suboptimal responses to antidepressants [73]. Low levels of vitamin D are linked to an increased risk of schizophrenia and depression [74].

Recognizing the pivotal role of diet in maintaining physical well-being is widespread, given the evident influence of dietary factors on cardiometabolic ailments, cancer, and premature death [75,76]. Yet, in addition to conventional food consumption, nutrients can also be ingested through supplements. Supplements are commonly utilized to do the following:

(a) Address dietary deficiencies or low measured plasma levels of specific nutrients to meet recommended intake levels;
(b) Provide specific nutrients at higher doses than typically found in a regular diet, aiming for potential physiological benefits;
(c) Offer nutrients in more readily absorbable forms for individuals with genetic variations or health conditions affecting nutrient absorption.

Supplements can be either artificially manufactured or derived directly from food sources, encompassing vitamins (e.g., folic acid and vitamin D); dietary minerals (e.g., zinc and magnesium); pre/probiotics (from specific gut-bacteria strains); polyunsaturated fatty acids (PUFAs), typically in the form of omega-3 fish oils; or amino acids (e.g., *N*-acetylcysteine and glycine). Presently, there is mounting academic and clinical interest in the utilization of nutrient supplements for managing various mental health disorders [77,78,79]. This burgeoning research interest partly stems from our evolving comprehension of the neurobiological foundations of mental illnesses, suggesting certain nutrients as potential supplementary treatments for several reasons [80]. Firstly, recent clinical investigations have revealed that many mental disorders coincide with heightened levels of oxidative stress and inflammation markers in both the central and peripheral systems [81,82,83,84]. Studies have indicated a correlation between the effectiveness of pharmacological and lifestyle interventions for mental illness and changes in these biomarkers [85,86]. Hence, the antioxidant and anti-inflammatory properties of certain nutrient supplements, such as *N*-acetylcysteine and omega-3 fish oils, suggest their potential efficacy in treating psychiatric conditions exacerbated by elevated inflammation and oxidative stress. Secondly, extensive data from large-scale studies indicate that psychotic and mood disorders are linked with significantly reduced serum levels of essential nutrients, including zinc, folate, and vitamin D [87,88,89,90,91]. Since these deficiencies appear to impact treatment response and clinical outcomes in affected populations, there is potential for nutrient supplementation to enhance outcomes [92,93]. Eicosapentaenoic acid (EPA) and docosahexaenoic acid (DHA) exert significant effects on the synthesis, release, receptor function, and storage of neurotransmitters during brain development and in neuropsychiatric disorders [94,95,96,97]. A national survey conducted in the United States found a notable association between EPA + DHA intake within the past 24 h and a 25% reduction in the prevalence of depressive symptoms among 10,480 adults [98]. A randomized controlled trial (RCT) by Rapaport et al. [99] demonstrated a positive impact of EPA in a subgroup of individuals with major depressive disorder (MDD) and elevated levels of inflammatory biomarkers. Another RCT involving depressed pregnant women showed that EPA supplementation, along with increased levels of estradiol during pregnancy, alleviated antenatal depression [100].

Studies examining serum magnesium levels in various mental disorders have been conducted, yet a definitive pathophysiological link between mental disorders and magnesium in patients has not been fully established. Some studies in animal models suggest a beneficial role of magnesium in reducing neuroinflammation [101,102]. The majority of studies in patients have primarily focused on depression, given magnesium’s involvement in core mechanisms of depressive pathophysiology, including inflammation and oxidative stress [103]. Among other proposed mechanisms, magnesium may alleviate depression by blocking the NMDA receptor, which is implicated in depression pathology [104]. Further research is needed to elucidate the role of magnesium in the pathophysiology or treatment of bipolar disorder and obsessive–compulsive disorder (OCD).

Folate deficiency is prevalent in nearly 30% of severely depressed inpatients, often accompanied by increased plasma homocysteine levels [105]. Some RCTs suggest that folate and vitamin B12 supplementation could be beneficial for the long-term management of specific populations with depressive symptoms. However, meta-analyses by Almeida OP et al. [106] highlighted the limited number and heterogeneity of available studies, suggesting the need for additional investigations.

The role of alpha-tocopherol (vitamin E) in the brain has been explored in animal models with deficiencies in vitamins C and E, revealing increased expression of inflammatory-related genes and potential neuroinflammation [107]. *In vitro* and preclinical *in vivo* studies in animal models demonstrated that alpha-tocopherol supplementation decreased neuroinflammation and oxidative stress [108].

Overall, the reviewed research data suggest a favorable role for vitamin D, EPA, DHA, magnesium, alpha-tocopherol, and folic acid, either alone or in combination with medications, in maintaining normal brain function and mental well-being, partly through the modulation of neuroinflammation. Further clinical trials are warranted to validate the findings and gain deeper insights into the mechanisms of action and optimal dosage range of these nutrients in promoting mental health and well-being. Addressing these challenges will be crucial for advancing our understanding of nutrient supplementation as a potential therapeutic strategy for mental health disorders.

## 4. The Impact of Selected Diets on Depression and Obesity

Overall diets play a crucial role in both depression and obesity. Diets emphasizing whole foods, fiber-rich foods, lean proteins, and healthy fats, while limiting processed foods, sugar, and saturated fats, are recommended. These include the Mediterranean diet and the DASH (Dietary Approaches to Stop Hypertension) diet. These diets provide essential nutrients and antioxidants that support brain health and reduce inflammation, contributing to better mood regulation. Due to the escalating prevalence of individuals opting for a vegetarian diet, we have also chosen to assess its effects on obesity and depression.

### 4.1. Mediterranean Diet

A study conducted on 289 nursing students in Spain from October 2022 to March 2023, ranging in age from 17 to 30, revealed a robust connection between adherence to the Mediterranean diet (MD) and mental well-being. Those who followed the MD exhibited significantly lower levels of anxiety and depression compared to their counterparts who did not adhere to the diet. Researchers propose that the protective effect of the MD against depression may stem from a combination of factors, including an adequate intake of omega-3 fatty acids and other natural unsaturated fats, antioxidants from olive oil and nuts, flavonoids, and other antioxidant compounds from fruits and vegetables, as well as abundant natural folate and other B vitamins [109].

Similarly, a study by Hwang et al. [110], which examined the link between adherence to the Mediterranean diet and depression among a representative sample of South Korean adults, found a reduced risk of depression among those who adhered closely to the diet. This association was significant across both genders, with individuals adhering more to the Mediterranean diet showing a 42–73% lower risk of depression. These findings align with previous research indicating a 40–45% lower risk of depression among individuals with higher Mediterranean diet scores [111]. Obesity’s adverse effects can be partially mitigated by significant weight loss, achievable through adherence to the Mediterranean diet (MD), particularly when coupled with a low-calorie approach and regular physical activity. Moreover, the MD’s composition has been linked to a notable reduction in dyslipidemia and a positive modulation of the gut microbiota and immune system, leading to a significant decrease in inflammatory markers—a common trigger for various obesity-related disorders [112].

A study investigating the impact of calorie-restricted MD on weight loss in overweight and obese individuals over 12 months found that MD yielded slightly better results in weight reduction compared to low-fat diets [113]. In another intervention spanning 8 weeks, which involved a calorie-restricted and protein-enriched MD, obese men awaiting laparoscopic gastrectomy experienced significant reductions in body weight and visceral fat [114]. Similarly, another study conducted over a 6-week period revealed that a hypocaloric MD was more effective in reducing body weight and preserving fat-free body mass (FFM) compared to high-protein diets in young individuals with sedentary lifestyles. Maintaining FFM is crucial for sustaining both short- and long-term weight loss benefits, as FFM is associated with a lower basal metabolism and decreased risk of developing sarcopenic obesity [115]

### 4.2. Vegetarian Diet

Emerging research indicates a potential link between vegetarian and vegan diets and an increased risk of depression, a prevalent mental health condition affecting millions worldwide. This connection has garnered significant attention, particularly given the growing popularity of plant-based diets globally [116]. A vegetarian diet primarily consists of plant-derived foods, such as vegetables, whole grains, legumes, nuts, seeds, and fruits, with some including non-meat animal products, like dairy, eggs, or honey. There are various classifications of vegetarians, including lacto-vegetarians, ovovegetarians, and lactoovovegetarians, each with specific dietary restrictions regarding meat, poultry, seafood, eggs, and dairy. On the other hand, vegans strictly avoid all animal products, including honey, dairy, eggs, and any animal-derived ingredients in processed foods [117].

The potential risk of depression associated with vegetarian and vegan diets stems from their inherent deficiencies in essential nutrients, such as vitamin B12 and long-chain omega-3 polyunsaturated fats (PUFAs), that are crucial for optimal brain function [118]. However, these diets may also offer protective benefits against depression due to their higher intake of fruits and vegetables, rich in antioxidants like vitamin C, vitamin E, and beta-carotene, which can combat inflammation in the brain linked to depressive symptoms [119]. Additionally, vegetarian diets have shown positive effects on metabolic health, including improvements in blood lipid profiles and body weight [120].

A comprehensive meta-analysis exploring the relationship between vegetarian diets and mental health revealed intriguing findings. It indicated that individuals following vegan or vegetarian diets may face a heightened risk of depression and exhibit lower anxiety scores compared to their non-vegetarian counterparts. Particularly noteworthy was the observation that, among populations aged under 26 years, vegetarians and vegans exhibited a greater susceptibility to various mental disorders compared to those adhering to non-vegetarian diets [121]. However, contrasting perspectives emerged from another meta-analysis, which failed to establish a clear association between vegetarian dietary patterns and depression [122].

Adding depth to this discourse, a recent systematic review and meta-analysis delved into the intricate link between vegetarian diets and depression. This analysis, encompassing 16 observational studies, spotlighted a significant trend. Among the nine studies incorporated into the meta-analysis, adherence to a vegetarian diet was linked with a striking 53% higher risk of depression in comparison to diets incorporating meat. Despite these compelling insights, the researchers underscored the need for further observational studies to provide conclusive evidence in this domain [123]. In turn, in the study of Walsh et al. [124], a notable correlation emerges between the quality of diet and depressive symptoms across various dietary patterns, encompassing omnivore, vegetarian, and vegan regimens. The findings reveal that superior diet quality is inversely associated with depressive symptoms, with vegan diets exhibiting the highest quality and the most pronounced protective impact against depression. These results underscore the potential of diet quality, irrespective of its meat- or plant-based composition, as a modifiable lifestyle element to mitigate the risk of depressive symptoms.

In summary, some outcomes suggest higher rates of depression associated with vegetarian and vegan diets, while others indicate beneficial effects or no association. Therefore, the researchers underscored the need for further observational studies to provide conclusive evidence in this domain.

### 4.3. DASH Diet

Perez-Cornago et al. [125] provides further evidence supporting the notion that maintaining a healthy diet could serve as a potent shield against depressive symptoms. Their prospective cohort study, encompassing 14,051 participants, unveiled compelling insights. Over an eight-year follow-up period, individuals adhering to a diet closely resembling the Dietary Approaches to Stop Hypertension (DASH) regimen exhibited a significantly reduced risk of depression [126]. These findings echo similar sentiments echoed across various age demographics. For instance, research involving adolescent girls highlighted the protective effects of a DASH-like diet against depression when compared to diets less aligned with its principles. Moreover, recent investigations by Fresán et al. [127] underscore the potential efficacy of both the Mediterranean and DASH diets in staving off depressive symptoms. Further reinforcing these observations, a cross-sectional study conducted among Iranian girls aged 12-to-18 years shed light on the benefits of strict adherence to a DASH-style diet. Notably, a lower risk of depression was associated with such dietary patterns, despite a substantial proportion of participants experiencing depressive symptoms. However, intriguingly, no discernible link emerged between DASH-diet adherence and reduced aggression levels in this cohort.

In summary, adopting a dietary pattern akin to the DASH diet, characterized by abundant whole grains, fruits, and vegetables; a moderate consumption of legumes and nuts; and the limited intake of red meat, processed meat, and sodium, has been linked to a reduced risk of depression. Given the widespread prevalence of depression and the compelling findings of this investigation, it appears plausible to regard the DASH diet as a potential strategy for preventing or managing depressive disorders [126]. Research indicates that the DASH diet is not only effective in lowering high blood pressure but also shows promise in weight management. A meta-analysis involving 13 studies revealed that adults adhering to the traditional DASH diet experienced a notable decrease in weight over a 24-week period compared to those following a typical Western diet. Moreover, the low-calorie variant of the DASH diet resulted in even more substantial weight loss when compared to other low-carbohydrate diets [128].

## 5. The Impact of Physical Activity and Its Lack on Obesity and Depressive Disorders

Globally, there has been a noticeable decline in physical activity levels compared to previous decades. While research indicates that participation in sports and recreational activities has remained stable or slightly increased, these pursuits represent only a fraction of overall physical activity. The decline in physical activity is primarily attributed to shifts in work-related, household, and transportation activities, influenced by economic development, technological progress, and societal shifts. Its contribution to everyday life is influenced not only by advances in civilization or the way leisure time is managed. Its extent also depends on the social status of individuals. With increasing social polarization and pauperization of a huge part of the population of highly developed countries, physical activity in the form of an intentional recreational activity is becoming less and less available. The need to function on a multi-job basis limits the possibility of life hygiene in this respect. Engaging in physical activity boosts an individual’s overall energy expenditure, aiding in maintaining energy balance and potentially leading to weight loss if calorie intake remains controlled. Additionally, regular physical activity is associated with decreased abdominal fat accumulation and overall body fat, which can help mitigate the progression of abdominal obesity. Moreover, participating in sports or exercise routines is linked to reduced feelings of depression and anxiety, with the resulting improved mood often serving as a motivating factor to sustain physical activity levels [129]. Aerobic exercise (AE), e.g., running, cycling, and rowing, are exercises that deplete oxygen in the muscles. Nevertheless, the oxygen consumption is sufficient to meet the energy requirements of the muscles and does not require energy to be obtained from another source. Aerobic exercise (AE) is a widely adopted approach for addressing obesity and overweight concerns. Numerous reviews and meta-analyses have scrutinized the impact of AE interventions on weight management. In a recent systematic review and meta-analysis conducted by Thorogood et al. [130], the efficacy of AE in individuals with obesity was assessed. Data from both 6-month and 12-month follow-ups were synthesized. The findings revealed that, after six months of exercise interventions, a modest weight loss of 1.6 kg was observed. Similarly, twelve-month AE interventions resulted in an average weight reduction of 1.7 kg and a decrease in waist circumference by 1.95 cm [130].

In Japan, a randomized controlled trial involving obese participants with at least two cardiovascular risk factors was conducted. The intervention group engaged in aerobic exercise 2–4 times per week for a duration of 6 months. Remarkably, this group exhibited significant reductions in body weight (−1.60 kg) and waist circumference (−1.8 cm), as well as improvements in triglyceride levels and *C*-reactive protein levels. Multiple studies suggested that aerobic exercise interventions lasting between 6 and 12 months, without calorie restriction, yield a 2–3% reduction in initial body weight. Notably, even a modest weight loss of less than 3%, attained through healthy lifestyle practices, including increased physical activity, offers health benefits comparable to more substantial weight loss without exercise [131]. Additionally, in 2009, Wu et al. [132] conducted a meta-analysis evaluating the impact of combined diet and exercise interventions lasting 6–12 months, compared to diet-only interventions, on weight loss among obese individuals. The findings indicated that combined diet and exercise interventions led to greater long-term weight loss compared to diet-only interventions [132].

Aerobic exercise stands out as a potent tool in combating obesity, known for its efficacy in improving overall fitness. It is advisable to engage in moderate-to-high-intensity aerobic activities that engage large muscle groups for optimal impact. To fully harness its benefits, aerobic exercise should be sustained over extended durations. Thus, aiming for at least 150-to-180 min of aerobic exercise per week can notably enhance fitness levels. Additionally, resistance exercise has demonstrated its effectiveness in contributing to weight management [133,134]. Kazmi et al. [135] conducted a study to assess the prevalence of obesity and overweight stemming from sedentary lifestyles in the urban slums of Lahore, Pakistan. Their research, involving 646 participants, spanned from September 2018 to September 2019. Results from the one-year follow-up revealed a noteworthy correlation between rising BMI and sedentary behavior, particularly among females who exhibited higher rates of overweight and obesity [135].

There is no denying that physical inactivity stands as the primary driver of the obesity epidemic, yet environmental factors can either exacerbate or mitigate this issue. A comprehensive meta-analysis involving 111,851 individuals revealed significant links between obesity, sedentary lifestyles, and a lack of physical activity [136].

Brandao et al. [137] demonstrated that physical activity can increase telomere length in obese individuals, independent of weight loss. Telomeres, protective protein complexes found at the ends of chromosomes, play a crucial role in limiting replication-induced chromosome shortening. Shortened telomeres have been associated with metabolic disorders, while maintaining and lengthening telomeres has been linked to longevity. In their study, Brandao et al. [137] worked with 13 premenopausal women with a BMI between 30 and 40 kg/m^2^. These participants underwent an 8-week physical activity intervention program consisting of 55 min of combined aerobic exercise and resistance training three times a week. Although there were no significant changes in body weight or BMI after the training program (*p* < 0.05), the researchers observed a 6% increase in lean mass (kg) (*p* < 0.05), an 8% increase in VO2 max (maximal oxygen uptake) (mL/kg/min) (*p* < 0.05), and a 2% reduction in waist circumference [137].

Two studies investigated the impact of physical activity on brain health indicators, regardless of weight loss, among individuals with obesity, focusing on changes in sleep patterns and depressive symptoms. These studies implemented combined aerobic and resistance training regimens [137]. Mendham et al. [138] examined whether physical training influenced sleep quality and depressive symptoms in women with obesity from low-socioeconomic-status communities. Participants were randomly assigned to either an exercise or control group, with no initial differences between the groups. The exercise group underwent 12 weeks of combined resistance and aerobic training (40–60 min, 4 days per week), while the control group maintained their regular diet and activity levels. The effects of the exercise intervention on sleep quality, depressive symptoms, sleep characteristics, peak oxygen consumption, and glucose metabolism were evaluated over the 12-week period. The intervention led to improvements in sleep quality (*p* < 0.001), sleep efficiency (*p* = 0.005), and severity of depressive symptoms (*p* = 0.002). These improvements were also associated with increased peak oxygen consumption and reduced sedentary time. Notably, depressive symptoms were positively correlated with peak oxygen consumption (*p* < 0.001), while improved sleep was linked to decreased sitting time (*p* = 0.018) [139]. This systematic review underscores the diverse physiological benefits of increased physical activity for individuals with obesity, highlighting the importance of emphasizing these health improvements rather than solely focusing on weight loss. However, due to the lack of comprehensive research on optimal practices, further long-term investigations in this field are warranted. Research investigating the correlation between physical activity and mental well-being consistently indicates that increased physical exertion correlates with a reduced risk of depression-related symptoms, particularly among younger individuals [139]. Animal studies have further illuminated this relationship, demonstrating that regular physical activity acts as a protective factor against the adverse effects of chronic stress on the structure of the prefrontal cortex. Notably, aerobic training has been shown to exert a beneficial morphological influence on neurons within the prefrontal cortex of animals subjected to chronic stressors. These findings underscore the pivotal role of both pre- and post-pubertal exercise in safeguarding against neuronal and behavioral abnormalities arising from distress [140]. Multiple studies underscore the significant role of physical activity in the management of depressive disorders. One notable study observed a positive impact of 24 weeks of interval training on alleviating symptoms associated with mild depressive disorders, as measured by the Beck Depression Inventory, among a cohort of 36 women without concurrent somatic ailments. Additionally, reductions in anxiety levels were observed among the participants [141]. Other studies have corroborated the efficacy of both interval training and aerobic exercise in preventing depression, particularly among adults diagnosed with major depressive disorder. In one study, 50 participants were divided into two groups, each consisting of 25 individuals, and underwent 12 training sessions. While the first group engaged in interval training, the second group participated in aerobic exercise. Upon a comparative analysis, no significant differences were observed between the two groups regarding changes in exercise motivation, perceived mood, cardiorespiratory fitness, or depressive symptoms. These findings suggest that individuals with depression may benefit from the flexibility to choose between different forms of physical activity based on their preferences and needs regarding the type and intensity of exercise [142]. This flexibility is especially pertinent for men with depression, who typically exhibit lower motivation levels for engaging in activities aimed at alleviating depressive symptoms compared to women [143].

## 6. The Effects of Physical Activity on the Modulation of Gut Microbiota Composition

Hampton-Marcell et al. [144] and Durk et al. [145] conducted studies that revealed variations in the Firmicutes-to-Bacteroidetes (F/B) ratio in response to exercise. In the first study, a decrease (↓ F/B) was observed with a reduction in training volume, whereas the second study indicated an increase (↑ F/B) attributed to an enhancement in VO2 max [144,145]. Additionally, Scheiman et al. [146] conducted a 2-week study involving 15 runners, which demonstrated an increase in *Veillonella bacteria* [146]. Kern et al. [147], in a study involving 88 obese and overweight individuals, observed a 5% rise in the Shannon index of microbiota after three months of regular intense training [147].

Zhong et al. [148] investigated the impact of aerobic and resistance exercise on fourteen women, revealing an increase in bacteria associated with anti-inflammatory effects (e.g., *Verrucomicrobia*), alongside a decrease in pro-inflammatory bacteria (e.g., *Proteobacteria*) [148]. O’Donovan et al. [149] explored the differences between the effects of dynamic and static exercise on microbiota changes among 37 elite Irish athletes. Their findings highlighted a significant dominance of *Streptococcus suis*, *Clostridium bolteae*, *Lactobacillus phage* LfeInf, and *Anaerostipes* in the moderate dynamic sports groups. Conversely, individuals engaged in high dynamic sports exhibited a significant presence of *Bifidobacterium animalis*, *Lactobacillus acidophilus*, *Prevotella intermedia*, and *F. prausnitzii* [149].

Moreover, in mice undergoing moderate long-term exercise, an increase in immunoglobulin A (IgA) production and a reduction in B and T-CD4 cells in the intestines were observed compared to sedentary mice. These findings suggest that exercise may strengthen the commensal microbiota, aiding in the defense against intestinal pathogens [150,151]. However, some studies have noted a decrease in the genus *F. prausnitzii* in exercising mice, potentially linked to pathologies in the fatty intestine [152]. This suggests that an imbalance in dietary intake relative to the body’s needs, coupled with exercise, could lead to alterations in the gut microbiota composition and barrier function of the intestinal mucosa [150,153].

These observations underscore the intricate relationship between nutritional status and exercise, particularly during early life, when the gut microbiota composition undergoes significant modifications. This shift is associated with appetite-related signaling, where serum leptin levels correlate positively with *Bifidobacterium* and *Lactobacillus populations* but negatively with *Bacteroides* spp. and *Prevotella* spp. levels. Conversely, ghrelin serum levels exert the opposite effects on these bacterial populations. Thus, early-life exercise may profoundly influence gut microbiota composition, fostering bacteria capable of inducing adaptive changes in host metabolism and optimizing brain function development [153,154].

## 7. Depression and Obesity and the Socio-Sociological Aspect

Depression and obesity represent significant public health challenges, as both have far-reaching implications for morbidity, mortality, and socioeconomic factors [150]. In the United States alone, the prevalence of major depressive disorder (MDD) approaches nearly 10% [155]. Depression stands as a leading cause of disability in developed nations. A recent review examining the impact of MDD revealed that individuals grappling with this condition experience functional impairment and a diminished quality of life comparable to, or even greater than, other prevalent chronic illnesses, such as diabetes, hypertension, and heart disease. Notably, effective treatment of depression yields comprehensive health enhancements encompassing mental, emotional, and social domains, along with an improved perception of health and quality of life [156,157]. Furthermore, depression correlates with increased absenteeism from work, amplifying the burden on workplaces. Conversely, alleviating depressive symptoms has been shown to enhance productivity in professional settings [157,158]. These findings underscore the multifaceted nature of depression’s impact on individuals and society at large, underscoring the imperative for effective interventions and support mechanisms to address this pervasive health issue. Today’s unprecedented prevalence of depression therefore carries serious social and systemic implications. The increased number of sufferers, with a significant proportion of young people, reduces their educational and occupational abilities. The consequences include untapped human potential, insufficient labor market participation, limited participation in public life, and the need to develop specialized medical services and reimbursement of medicines. Other consequences of the silent depression pandemic are reduced social mobility and negative demographic consequences, which are not insignificant for the maintenance of social security systems based on the principle of intergenerational solidarity.

Likewise, obesity stands as a pervasive global health challenge. In the United States, the prevalence of obesity hovers around 30%, a statistic that has shown a consistent upward trajectory in recent years [159]. This persistent rise in obesity rates underscores the urgency of addressing this issue on a societal level. Beyond its widely recognized medical complications, obesity also exerts profound psychiatric ramifications [160]. Moreover, it significantly diminishes quality of life, contributes to cognitive impairment, and is linked to premature mortality and reduced life expectancy. Particularly concerning for women, obesity can detrimentally affect reproductive health, serving as a confirmed factor in female infertility. Socially, individuals grappling with obesity encounter barriers in the job market, tend to retire earlier, and often rely more heavily on disability benefits due to increased absenteeism and decreased productivity [161]. Extensive epidemiological studies and meta-analyses have consistently underscored the association between depression and obesity as a prevalent comorbidity [162]. This intricate interplay between mental and physical health highlights the necessity for holistic approaches to address obesity and its multifaceted impacts on individuals and society.

## 8. Conclusions

The coexistence of obesity and depressive disorders represents a prevalent comorbidity, intertwining distinct yet interrelated pathophysiological pathways. Obesity exerts a direct and potentially toxic impact on brain function, exacerbating the complexity of mental health issues. This convergence of obesity and depressive disorders is tightly linked to detrimental health outcomes and subsequent social ramifications. Dysregulation in the composition of gut microbiota has been linked to mood disorders and depression, suggesting a potential therapeutic avenue through probiotic interventions. Incorporating strains with documented effects, alongside prebiotic substances and a balanced diet, holds promise for individuals grappling with such disorders. Encouraging findings from existing research underscore the need for robust clinical trials to evaluate the therapeutic potential of microbiota modulation, shedding light on the intricate mechanisms linking gut microbiota to mental well-being. Similarly, in the realm of obesity, mounting evidence underscores the detrimental impact of gut microbiota imbalance and compromised gut barrier function. Probiotics, encompassing psychobiotics and prebiotics, have emerged as valuable tools in mitigating metabolic dysregulation among overweight or obese individuals. Moreover, they exhibit potential in enhancing intestinal integrity, thereby reducing systemic inflammation risk. Notably, probiotics serve as a source of short-chain fatty acids (SCFAs), pivotal in orchestrating anti-inflammatory processes within the body. Strains that directly affect the activation of neural pathways, thereby reducing stress in the body, include strains such as *Lactobacillus casei Shirota*, *Lactobacillus fermentum* NS8 and NS9, *Lactobacillus rhamnosus* JB-1, *Lactobacillus gasseri* OLL2809, *Lactobacillus helveticus* Rosell-52, *Lactococcus lactis* W19 and W58, *Lactobacillus acidophilus* W37, *Lactobacillus brevis* W63, *Bifidobacterium longum* Rosell-175, *Bifidobacterium longum* 1714, *Bifidobacterium longum* NCC3001, *Bifidobacterium bifidum* W23, *Lactobacillus plantarum* 299v, and *Bifidobacterium lactis* W52.
